# Ophthalmological findings in children with non-syndromic craniosynostosis: preoperatively and postoperatively up to 12 months after surgery

**DOI:** 10.1136/bmjophth-2020-000677

**Published:** 2021-04-25

**Authors:** Evangelia Ntoula, Daniel Nowinski, Gerd Holmstrom, Eva Larsson

**Affiliations:** 1 Department of Neuroscience/Ophthalmology, Uppsala University, Uppsala, Sweden; 2 Department of Surgical Sciences/Plastic Surgery, Uppsala University, Uppsala, Sweden

**Keywords:** child health (paediatrics), epidemiology, muscles, optics and refraction, vision

## Abstract

**Aims:**

Craniosynostosis is a congenital condition characterised by premature fusion of one or more cranial sutures. The aim of this study was to analyse ophthalmic function before and after cranial surgery, in children with various types of non-syndromic craniosynostosis.

**Methods:**

Children referred to Uppsala University Hospital for surgery of non-syndromic craniosynostosis were examined preoperatively. Visual acuity was measured with Preferential Looking tests or observation of fixation and following. Strabismus and eye motility were noted. Refraction was measured in cycloplegia and funduscopy was performed. Follow-up examinations were performed 6–12 months postoperatively at the children’s local hospitals.

**Results:**

One hundred twenty-two children with mean age 6.2 months were examined preoperatively. Refractive values were similar between the different subtypes of craniosynostosis, except for astigmatism anisometropia which was more common in unicoronal craniosynostosis. Strabismus was found in seven children, of which four had unicoronal craniosynostosis.

Postoperatively, 113 children were examined, at mean age 15.9 months. The refractive values decreased, except for astigmatism and anisometropia in unicoronal craniosynostosis. Strabismus remained in unicoronal craniosynostosis. Two new cases with strabismus developed in unicoronal craniosynostosis and one in metopic, all operated with fronto-orbital techniques. No child had disc oedema or pale discs preoperatively or postoperatively.

**Conclusion:**

Ophthalmic dysfunctions were not frequent in children with sagittal craniosynostosis and preoperative ophthalmological evaluation may not be imperative. Children with unicoronal craniosynostosis had the highest prevalence of strabismus and anisometropia. Fronto-orbital techniques used to address skull deformity may be related to a higher prevalence of strabismus postoperatively.

**Key messages**

**What is already known about this subject?**

- Syndromic craniosynostosis is associated with high prevalence of ophthalmic abnormalities.
- Ophthalmological manifestation in non-syndromic craniosynostosis is less severe and therefore less described.
- Strabismus, refractive errors and amblyopia have been reported, especially in unicoronal craniosynostosis.

**What are the new findings?**

- Ophthalmological dysfunctions were not frequent in children with sagittal craniosynostosis.
- Fronto-orbital techniques used to address skull deformity may be related to a higher prevalence of strabismus postoperatively.

**How might these results change the focus of research or clinical practice?**

- Children with sagittal craniosynostosis might not need to undergo a routine ophthalmological examination preoperatively, as the ones with unicoronal and metopic synostosis do.
- Postoperative ocular muscle imbalance causing strabismus necessitates the development of new operating strategies.
- Future examinations at preschool and school age will further elucidate the long-term ophthalmological effects and shape development of the follow-up protocols in children with non-syndromic craniosynostosis.

## Introduction

Craniosynostosis is a complex congenital condition characterised by premature fusion of one or more cranial sutures. It results in a restricted skull growth pattern across the fused suture and compensatory growth in a parallel direction, leading to characteristic cranial deformations. The care of craniosynostosis is conducted by multidisciplinary teams with longitudinal follow-up. Most children with non-syndromic craniosynostosis undergo surgery to normalise head shape and prevent intracranial hypertension.

In the majority of patients, craniosynostosis involves a single suture and is non-syndromic. More rarely, the suture fusion is part of a craniofacial or rare genetic syndrome.^1^ The most common single-suture craniosynostosis, which accounts for almost half of all cases, is sagittal synostosis (scaphocephaly), with an incidence of 1:5000 births. This is followed, in descending order of frequency, by metopic synostosis (trigonocephaly), unilateral coronal synostosis (anterior plagiocephaly) and unilateral lambdoid synostosis (posterior plagiocephaly). In syndromic craniosynostosis, multiple suture involvement is common, as are malformations of the cranial base and midface skeleton. Syndromic craniosynostosis is associated with severe orbital deformity and a resulting high prevalence of ophthalmic abnormalities such as refractive errors, strabismus, eyelid abnormalities, proptosis and exposure keratitis. Papilloedema and/or optic atrophy related to increased intracranial pressure may cause amblyopia and visual impairment.^2 3^

Ophthalmic manifestations in non-syndromic craniosynostosis are less severe and less well described. Nonetheless, visual dysfunctions, strabismus, refractive errors and amblyopia have been reported.^4–6^

Orbital anatomy may be affected in the different forms of single-suture craniosynostosis, but to a lesser degree than in the syndromes. Moreover, the surgical treatment in metopic and coronal synostosis entails periorbital dissection and orbital skeleton rearrangement, with risk of iatrogenic visual dysfunction.

The aim of this study was to comprehensively analyse any ophthalmic abnormalities preoperatively, as well as any immediate effects from craniosynostosis surgery during the early postoperative course, in children treated for various types of non-syndromic craniosynostosis.

## Materials and methods

The patients included in this study had been referred to Uppsala Craniofacial Centre, Uppsala University Hospital, Sweden, between May 2012 and June 2018. This is one of two licensed national reference centres for paediatric craniofacial surgery and was established in 2012. Infants with non-syndromic craniosynostosis, confirmed through CT scan, were examined preoperatively by one of three experienced orthoptists and paediatric ophthalmologists, respectively, as part of a multidisciplinary craniofacial assessment, following a specially designed protocol. All patients with suspected syndromic malformations, other malformations and with synostosis of the coronal suture(s) were genetically analysed, on a routine basis. Children with craniosynostosis syndromes, such as Crouzon, Apert, Pfeiffer and Saethre-Chotzen syndromes, complex craniosynostosis or other rare genetic syndromes, confirmed through genetic analysis, were excluded. One child with Muenke syndrome was included because of presentation with only unicoronal craniosynostosis but lack of other craniofacial malformations.

### Ophthalmological examinations

Visual acuity (VA) was measured with the Preferential Looking test (PL), (Teller Acuity Cards or Cardiff Cards), monocularly and binocularly, or in children who were too young to cooperate, with observation of fixation and following. This was assessed by observing the ability of the infant to fix and follow a 5 cm target at a distance of 30 cm, and looking for quality of pursuit and asymmetry between the eyes. In older children, logMAR optotypes (Lea or HVOT) were used.^7 8^ The orthoptic evaluation was conducted with cover–uncover test and when not possible, by evaluation of symmetry of corneal reflexes, that is, the Hirschberg test. Abnormalities in eye motility were noted. The anterior segment was examined. The refraction was measured in cycloplegia after instillation of eye drops including cyclopentolate 0.5% and phenylephrine 0.5% in children under 1 year of age, otherwise cyclopentolate 1.5% and phenylephrine 0.85%. The spherical component and astigmatism were noted and the spherical equivalent was calculated. Astigmatism and anisometropia were considered significant if greater than 1.00 dioptre (D). Funduscopy was performed through dilated pupils.

At 6–12 months after surgery, medical records regarding visual outcome, refraction, funduscopy and orthoptic measurements were retrieved from the children’s local hospitals. The follow-up examinations were performed by well-experienced orthoptics and paediatric ophthalmologists in accordance with the designed protocol.

### Statistical methods

Descriptive statistics were analysed using SPSS V.26 (IBM Corp). Mean and median values were calculated as were ranges. For further analyses, SAS software V.9.4 (SAS Institute) was used. For variables with binary (yes/no) outcome, exact logistic regression models to analyse the effect of type on the outcomes were used. For other variables, ordinal (proportional odds) logistic regression was used. Types of craniosynostosis as well as gender were analysed as explanatory variables. In order to evaluate the impact of age on the effect of type of craniosynostosis on the outcomes, we added age to the logistic regression models including type of craniosynostosis. For outcome variables measured before surgery, age before surgery was used in the analysis and for those measured after surgery, age after surgery was used.

Due to the exploratory nature of the study, nominal p values are reported without adjustment for multiplicity. A p value of <0.5 was regarded as statistically significant.

## Results

The study included 122 (93 boys) children. Mean and median ages at preoperative ophthalmological examination were 6.2 months and 4.7 months, respectively (range 0.6–40.5 months). Thirty-four (28%) of the 122 patients were less than 3 months old, 47 (39%) were between 3 and 6 months, 32 (26%) between 6 and 12 months and 9 (7%) were at or above 12 months of age.

The different types of craniosynostosis, together with the ages at first examination and at surgery, are given in table 1.

**Table 1:** Number and fraction (%) of 122 children included in the study divided by type of craniosynostosis, together with sex, age at preoperative examination, age at time of surgery and operation technique used

	Number (%)	Sex m:f	Age at first examination in months	Age at surgery in months	Operation technique
Sagittal	84 (69)	70:14	4.3 (0.6–36.8)	4.6 (3.2–36.9)	H-craniectomy 71/84 (85%) Cranial vault remodelling 13/84 (15%)
Metopic	22 (18)	17:5	4.5 (1.2–11.2)	8.0 (6.1–11.2)	Fronto-orbital remodelling 22/22
Unicoronal	16 (13)	6:10	8.0 (1.9–40.5)	10.3 (7.8–42.6)	Bilateral fronto-orbital advancement 16/16

Median values and ranges are given for ages.

f, female; m, male.

The mean/median age at the time of surgery was 7.7/6.0 months (range 3.2–42.6 months). Of 84 children with sagittal synostosis, 71 (85%) were operated before 6 months of age with the so-called H-craniectomy or Renier technique (extended strip craniectomy with parietal outfracture) and 13 (15%) at a later timepoint with cranial vault remodelling due to later primary diagnosis and referral. Metopic craniosynostosis was addressed with fronto-orbital remodelling and unicoronal craniosynostosis with bilateral fronto-orbital advancement (see table 1).

Data on ophthalmological follow-ups at 6–12 months after surgery were available for 113 of 122 children (84 boys): 17 examined at the Department of Ophthalmology, Uppsala University Hospital, and 96 children at other hospitals (records retrieved). The mean/median age at follow-up was 15.9/14.5 months (range 5.7–47.5 months). The different types of craniosynostosis and age at follow-up examination are presented in table 2.

**Table 2:** Number and fraction (%) of 113 children who were followed up at 6–12 months postoperatively divided by type of craniosynostosis, together with sex and age at postoperative examination

	Number (%)	Sex m:f	Age at examination (months)
Sagittal	78 (69)	62:16	13.7 (5.7–47.5)
Metopic	20 (18)	16:4	15.8 (10.6–20.7)
Unicoronal	15 (13)	6:9	21.0 (13.5–44.1)

Median values and ranges are given for age.

f, female; m, male.

There were no differences regarding refraction or strabismus between boys and girls.

### Sagittal craniosynostosis

In 67 of 84 children, VA was assessed with PL tests, of which 4 only binocularly, and was found normal for their age according to the manuals. Twelve children were tested with fixation and following, of which three only binocularly, and their visual behaviour was considered normal. Five children, all under 2 months of age, could not comply with any method of VA testing.

Postoperatively, information about VA was available for 59 of 84 children. Fifty-eight children could be examined with PL tests. All were considered to have normal vision for their age. One child with late diagnosed craniosynostosis had normal vision in both eyes, as assessed with Lea optotypes (VA ≤0.2 logMAR (≥0.63 Snellen decimal)), both preoperatively (at 3 years of age) and 6 months postoperatively.



Refraction was assessed in 73 of 84 right eyes (REs) and 74 of 84 left eyes (LEs) preoperatively, and in 67 of 78 REs and LEs postoperatively. Preoperatively, the mean/median spherical equivalent (SE) was +2.61/+2.50 D (range 0.00 D to +7.00 D) in REs and +2.64/+2.50 D (range 0.00 D to +6.50 D) in LEs. Postoperatively, the mean/median SE was +1.74/+1.75 D (range −1.25 D to +4.75 D) in the REs and +1.72/+1.5 D (range −1.87 D to +4.25 D) in the LEs, respectively. The median preoperative values of spherical components and prevalence rates of astigmatism ≥1 D and anisometropia of spherical component and astigmatism ≥1 D are given in table 3 and postoperative values in table 4.

**Table 3:** Preoperative ophthalmological examination

	Refraction spherical RE	Refraction spherical LE	Astigmatism ≥1 D RE	Astigmatism ≥1 D LE	Anisometropia spherical ≥1 D	Anisometropia astigmatism ≥1 D
Total	+2.75 D (0 to +7.00)	+3.00 D (0 to +6.50)	43/107 (40.0%)	47/108 (43.5%)	5/107 (4.7%)	8/107 (7.5%)
Sagittal	+3.00 D (+1.00 to +7.00)	+3.00 D (+1.00 to +6.50)	29/73 (39.7%)	34/74 (45.9%)	1/73 (1.4%)	2/73 (2.7%)
Metopic	+3.00 D (+1.00 to +5.00)	+3.00 D (0 to +5.00)	9/20 (45.0 %)	7/20 (35.0%)	1/20 (5.0%)	1/20 (5.0%)
Unicoronal	+2.38 D (0 to +4.00)	+2.62 D (0 to +4.50)	5/14 (35.7%)	6/14 (42.8%)	3/14 (21.4%)	5/14 (35.7%)

Median values (range) of spherical component and prevalence rates of astigmatism ≥1 D and anisometropia of spherical component and astigmatism ≥1 D in 107 REs and 108 LEs.

D, dioptre; LE, left eye; RE, right eye.

**Table 4:** Postoperative ophthalmological examination

	Refraction spherical RE	Refraction spherical LE	Astigmatism ≥1 D RE	Astigmatism ≥1 D LE	Anisometropia spherical ≥1 D	Anisometropia astigmatism ≥1 D
Total	+2.00 D (−0.75 to +5.50)	+2.00 D (−1.25 to +5.50)	20/89 (22.5%)	24/89 (27.0%)	7/89 (7.9%)	6/89 (6.7%)
Sagittal	+2.00 D (−0.75 to +5.50)	+2.0 D (−1.25 to +5.00)	16/67 (23.9%)	17/67 (25.4%)	2/67 (2.9%)	2/67 (3.0%)
Metopic	+2.00 D (+1.00 to +5.00)	+1.50 D (+1.0 to +5.50)	2/13 (15.4 %)	3/13 (23.1%)	1/13 (7.7%)	1/13 (7.7%)
Unicoronal	+2.00 D (+1.00 to +4.25)	+2.50 D (+1.0 to +5.25)	2/9 (22.2%)	4/9 (44.4%)	4/9 (44.4%)	3/9 (33.3%)

Median values (range) of spherical component and prevalence rates of astigmatism ≥1 D and anisometropia of spherical component and astigmatism ≥1 D in 89 REs and LEs

D, dioptre; LE, left eye; RE, right eye.

Strabismus (exotropia) was found in three children all under 3 months of age at the preoperative examination. In all cases, the exotropia disappeared after surgery. In five children, all examined preoperatively under 1 ½ months of age, strabismus could not be assessed. None of them was found to have strabismus postoperatively. No new cases of strabismus were found.

### Metopic craniosynostosis

Preoperatively, 18 of 22 children were assessed with PL tests, of which 1 only binocularly and had normal vision for their age. Three children could be tested only with fixation and following of which two only binocularly and visual behaviour was considered normal. One child, under 2 months of age, could not comply with any method of vision testing. Postoperatively, 20 children were examined with PL tests. One child was found to have subnormal VA. This child was prescribed eyeglasses due to high hypermetropia.

Refraction was assessed in 20/22 REs and LEs, preoperatively, and 13/20 REs and LEs, postoperatively. Data are presented in tables 3 and 4, respectively. The mean/median SE was +2.20/+2.38 D (range +0.50 D to +5.00 D) in the REs and +2.14/+2.38 D (range −0.50 D to +5.00 D) in the LEs, preoperatively. Postoperatively, the mean/median SE was +1.83/+1.88 D (range +0.75 D to +4.75 D) in the REs and +1.92/+1.5 D (range +0.63 D to +5.25 D) in the LEs.

No child had strabismus preoperatively. However, one was found to have esotropia postoperatively.

### Unicoronal craniosynostosis

Preoperatively, 14 of 16 children were assessed with PL tests (one only binocularly), and 3 had subnormal vision for their age in one eye. One 2-month-old child could be tested only with fixation and following, and visual behaviour was considered normal. One child with late diagnosed craniosynostosis at 3 years of age had normal vision in both eyes preoperatively and postoperatively, as assessed with Lea optotypes: VA ≤0.2 logMAR (≥0.63 Snellen decimal).

Postoperatively, 11 of 15 children could be examined with PL tests, and 2 were found to have subnormal vision in one eye. Two children could not comply with any method of VA test. Information about VA was not available in 1 of 15 children.

Refraction was assessed in 14/16 REs and LEs preoperatively and in 9/15 REs and LEs postoperatively. Data are presented in tables 3 and 4, respectively. The mean/median SE was +1.90/+1.75 D (range −0.25 D to +4.00 D) in the REs and +1.95/+2.25 D (range −0.25 D to +4.00 D) in the LEs preoperatively. Postoperatively, the mean/median SE was +1.63/+1.75 D (range +0.50 D to +2.50 D) in the REs and +1.78/+2.00 D (range −0.50 D to +3.12 D) in the LEs.

**Figure 1:**
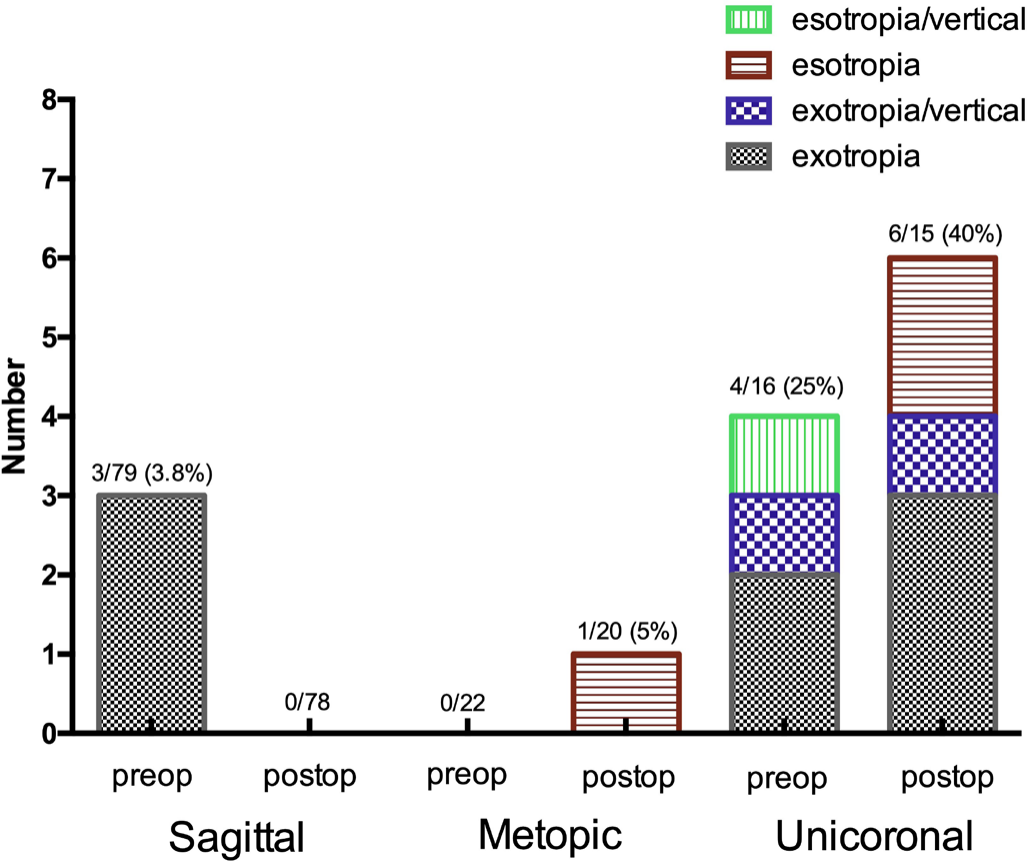
Prevalence of strabismus preoperatively and postoperatively in sagittal, unicoronal and metopic non-syndromic craniosynostosis.

Four children had strabismus preoperatively—three exotropia and one esotropia—which remained postoperatively. Two new cases of strabismus were found (figure 1). In three children, 
an overaction of the inferior oblique muscle was also noted postoperatively.

Regarding occlusion therapy and eyeglasses to prevent amblyopia, three children with unicoronal synostosis, all under 9 months of age, were prescribed occlusion before surgery, because of anisometropia, or a combination of anisometropia and strabismus. Postoperatively, they still had anisometropia and were prescribed eyeglasses. Three children developed a refractive error and anisometropia postoperatively and were also prescribed eyeglasses.

The anterior segment was considered normal in all children with craniosynostosis. No infant had disc oedema or pale discs preoperatively or postoperatively.

### Comparison of craniosynostosis

When comparing refraction, adjusted for age, there was a difference regarding spherical anisometropia ≥1 D preoperatively (p=0.05) and postoperatively (p=0.02) (see tables 3 and 4). Likewise, there was a difference in anisometropia of astigmatism ≥1 D preoperatively (p=0.01) and postoperatively (p=0.03) (tables 3 and 4). No statistical differences were found concerning the spherical component or astigmatism in REs nor LEs between the groups preoperatively and postoperatively. Regarding strabismus, there was a difference among subtypes of craniosynostosis both preoperatively (p=0.02) and postoperatively (p<0.001) (see figure 1, children with unicoronal synostosis having higher prevalence).

## Discussion

In this study of 122 children treated for various types of non-syndromic craniosynostosis, ophthalmic dysfunctions preoperatively and postoperatively appeared to be rare in those operated for sagittal synostosis. The children with unicoronal craniosynostosis had the highest prevalence of strabismus and anisometropia. Three children operated with fronto-orbital techniques to address their skull deformity, developed strabismus postoperatively.

Most ophthalmological studies on craniosynostosis concern the syndromes, in which ophthalmological problems are frequent.^2 3^ Further, the strabismus and refractive errors seen in unicoronal synostosis are well established and surgical procedures tailored to reduce strabismus have been suggested.^9^ There are, however, few studies on the ophthalmological findings before and after surgery in all subtypes of non-syndromic craniosynostosis. Most studies describe the outcome in metopic and unicoronal craniosynostosis.^10–15^ Vasco *et al*^5^ followed 29 children, including all subtypes, up to 1 year after surgery and concluded that abnormalities of visual function were more frequent preoperatively and that there was an improvement after surgery, though implying that it could be a sign of delayed visual maturation. Chieffo *et al*^16^ recently reported on a large cohort of 142 children with non-syndromic craniosynostosis and found high rates of neuro-ophthalmological or neuro-visual deficits at the time of diagnosis, improving 1 year after surgery.

There is a consensus among craniofacial surgeons that surgical intervention before 1 year of age is preferred, to optimise correction of deformity and prevent any harmful effects on brain development. However, there is a large variability in treatment protocols between centres. Sagittal craniosynostosis can be treated at an early stage, between 3 and 6 months of age, with relatively less invasive procedures—such as the extended craniectomy used at our centre. Surgery in children older than 6 months of age is more extensive, typically involving remodelling of the forehead. Metopic and unicoronal synostosis are typically operated with fronto-orbital remodelling/advancement between at 6 and 18 months of age (in order to reduce the risk of recurrence). The varying timepoints for initial referral to our unit and subsequent surgery between the different types of craniosynostosis were the reason why the children in our cohort underwent ophthalmological examination at different ages (see table 1). Different ages of surgery as well as of preoperative and postoperative examinations may explain different findings among studies. In addition, more immature infants are usually more difficult to examine and this might also have an impact on the ophthalmological outcome.

The refraction of the eye varies with the age of a child. Most neonates are hypermetropic, usually in combination with astigmatism, which diminishes with growth.^17^ In this study, the preoperative refractive values were rather similar between the different subtypes of craniosynostosis—with relatively high rates of hypermetropia and astigmatism, as expected—except for anisometropia which was more common in unicoronal craniosynostosis (table 2). This is in agreement with other studies in the literature.^12–15^ It has been speculated that the orbital abnormalities in unicoronal craniosynostosis may have an impact on corneal curvature causing astigmatism.^12 14^

At follow-up, the anisometropia remained in children with unicoronal craniosynostosis, whereas it decreased in the other subtypes. This was in contrast to the study by Vasco *et al*,^5^ in which no changes were found in refraction before and after surgery. Chieffo *et al*^16^ did not report on the refractive outcome.

The prevalence of strabismus across the general population is reported to be approximately 2%.^18^ In our cohort, the prevalence was higher, both preoperatively and postoperatively, particularly in unicoronal synostosis. This is in agreement with previous studies.^19^ Regarding unicoronal craniosynostosis, Gupta *et al*^15^ found exotropia as the only type of deviation in 3 of 45 children. Denis *et al*^20^ found esotropia and vertical strabismus in 4 of 21 children. Chieffo *et al*^16^ reported exotropia in 1 of 17, as well as 3 with disturbance of gaze elevation. Macintosh *et al*^13^ found strabismus in 34 of 55 children, in which esotropia with a vertical component was most common. In our cohort, 4 of 16 children had strabismus preoperatively: exotropia or esotropia or exotropia combined with vertical strabismus (figure 1). After surgery, strabismus remained in all children with unicoronal craniosynostosis, although the vertical component disappeared in one. Two new cases of strabismus developed after surgery (figure 1). No child with metopic craniosynostosis in our group had strabismus preoperatively, but one patient developed strabismus postoperatively. This is in agreement with other studies, where low prevalence rates of strabismus in metopic craniosynostosis are seen.^10 16 21^ There are reports on iatrogenic induction of strabismus from the fronto-orbital surgery itself.^22 23^ This phenomenon is most probably caused by the periorbital dissection including release of the trochlea and the rearrangement of the orbital skeleton involved in fronto-orbital remodelling procedures. This emphasises the importance of advancing surgical techniques towards less invasive procedures. Indeed, it has been proposed that earlier fronto-orbital surgery with less invasive methods could reduce the risk of iatrogenic strabismus.^9^

Regarding sagittal craniosynostosis and strabismus, less is reported. In the study by Chieffo *et al*,^16^ the authors found no strabismus preoperatively. Postoperatively, 3 new cases of 45 had developed exotropia. However, the age at examination or type of surgery was not reported. In our cohort, exodeviation was found in 3 of 79 children preoperatively, all under 3 months of age. All cases resolved postoperatively. We hypothesise that this resulted from the natural progression of ocular development rather than being a result of surgical intervention, as it is common for neonates to exhibit some degree of exodeviation, which resolves over time.^24^ As mentioned above, at our centre, children with sagittal synostosis were usually operated within the first 6 months of life; an early preoperative ophthalmological evaluation was therefore performed in these cases.

The increased risk of amblyopia in children with unicoronal synostosis has been reported by other authors.^12 13^ In the present study, VA was generally considered to be normal for age in the total group, in line with the study by Vasco *et al*,^5^ although cases of monocular amblyopia might have been missed in the children assessed only binocularly. Nevertheless, amblyopia must be prevented in cases of high refractive errors or strabismus. In our cohort, only children with unicoronal or metopic craniosynostosis had to be treated and were prescribed occlusion and eyeglasses at follow-up, whereas no child with sagittal craniosynostosis was.

Regarding optic nerve swelling or pale discs as a sign of elevated intracranial pressure, the results vary between different studies. In this study, no cases were found on funduscopy preoperatively. This was in contrast to the study by Chieffo *et al*,^16^ in which the authors found pallor of the optic discs in 51 of 142 children before surgery, for all non-syndromic craniosynostoses combined. Bennett *et al*^19^ found only 1 in 172 children with papilloedema preoperatively and none postoperatively, without reporting by type of craniosynostosis. We hypothesise that the subjectivity of funduscopy for evaluation of optic atrophy or papilloedema is one of the causes of the differing results. Further, the low sensitivity of funduscopy (around 20%), as a screening tool for detecting elevated intracranial pressure, has been discussed in literature.^25–28^

In this study, no child developed optic nerve changes within 6–12 months postoperatively. In the study by Chieffo *et al*,^16^ 4 (two sagittal, two metopic) of the 51 children had persistence of optic disc pallor after surgery, and no new cases were reported.

The strength of this prospective study was the large cohort, with all children being examined preoperatively at our department by a multidisciplinary team. All examinations followed a designed protocol and were performed by an orthoptist and a paediatric ophthalmologist, to obtain reliable data and comprehensive assessments. One limitation was the differing ages at the time of preoperative examination, as age is important when evaluating ophthalmological outcome in children. This variability was due to variable timing of initial referral and surgery, since the preoperative examinations were scheduled at initial assessment or just prior to surgery. However, in our analyses, we adjusted for age at examination. Another limitation was that the follow-up examination for the majority of the patients was performed at the referring hospital, and not by the craniofacial team orthoptist and ophthalmologist. However, these follow-up examinations were performed in accordance with instructions disseminated from our unit to all ophthalmological units within our referral network.

In conclusion, children with single-suture sagittal craniosynostosis appeared to have low prevalence of ophthalmic dysfunctions. Therefore, they might not need to undergo a routine ophthalmological examination preoperatively, as the ones with unicoronal and metopic synostosis do. Unicoronal craniosynostosis had the highest prevalence of strabismus and anisometropia, manifestations correlated with the orbital dysmorphology. Remodelling techniques used for the correction of skull deformity can affect the anatomy of the orbit, leading to postoperative ocular muscle imbalance, which necessitates the development of new operating strategies. Future examinations at preschool and school age will further elucidate the long-term ophthalmological effects and shape development of the follow-up protocols in children with non-syndromic craniosynostosis.

## Data Availability

Data are availble upon request.
